# The cost-effectiveness of hospital-based telephone coaching for people with type 2 diabetes: a 10 year modelling analysis

**DOI:** 10.1186/s12913-016-1645-6

**Published:** 2016-09-27

**Authors:** J. E. Varney, D. Liew, T. J. Weiland, W. J. Inder, G. A. Jelinek

**Affiliations:** 1 Department of Gastroenterology, Faculty of Medicine, Nursing and Health Sciences, Monash University, Level 6, The Alfred Centre, 99 Commercial Road, Melbourne, 3004 Australia; 2 Department of Epidemiology and Preventive Medicine, Faculty of Medicine, Nursing and Health Sciences, Monash University, Level 6, The Alfred Centre, 99 Commercial Road, Melbourne, 3004 Australia; 3 St Vincent’s Hospital, Melbourne and University of Melbourne, Melbourne, Australia; 4 Princess Alexandra Hospital and The University of Queensland, 199 Ipswich Road, Woolloongabba, QLD 4102 Australia; 5 University of Melbourne, Melbourne, Australia

**Keywords:** Type 2 diabetes, Telephone coaching, Cost-effectiveness

## Abstract

**Background:**

Type 2 diabetes (T2DM) is a burdensome condition for individuals to live with and an increasingly costly condition for health services to treat. Cost-effective treatment strategies are required to delay the onset and slow the progression of diabetes related complications. The Diabetes Telephone Coaching Study (DTCS) demonstrated that telephone coaching is an intervention that may improve the risk factor status and diabetes management practices of people with T2DM. Measuring the cost effectiveness of this intervention is important to inform funding decisions that may facilitate the translation of this research into clinical practice. The purpose of this study is to assess the cost-effectiveness of telephone coaching, compared to usual diabetes care, in participants with poorly controlled T2DM.

**Methods:**

A cost utility analysis was undertaken using the United Kingdom Prospective Diabetes Study (UKPDS) Outcomes Model to extrapolate outcomes collected at 6 months in the DTCS over a 10 year time horizon. The intervention’s impact on life expectancy, quality-adjusted life expectancy (QALE) and costs was estimated. Costs were reported from a health system perspective. A 5 % discount rate was applied to all future costs and effects. One-way sensitivity analyses were conducted to reflect uncertainty surrounding key input parameters.

**Results:**

The intervention dominated the control condition in the base-case analysis, contributing to cost savings of $3327 per participant, along with non-significant improvements in QALE (0.2 QALE) and life expectancy (0.3 years).

**Conclusions:**

The cost of delivering the telephone coaching intervention continuously, for 10 years, was fully recovered through cost savings and a trend towards net health benefits. Findings of cost savings and net health benefits are rare and should prove attractive to decision makers who will determine whether this intervention is implemented into clinical practice.

**Trial registration:**

ACTRN12609000075280

**Electronic supplementary material:**

The online version of this article (doi:10.1186/s12913-016-1645-6) contains supplementary material, which is available to authorized users.

## Background

Considerable economic burden is imposed by type 2 diabetes (T2DM) [1], which is increasing in prevalence. Interventions that improve risk factor status and clinical guideline adherence may prevent complications and reduce the healthcare costs associated with T2DM. Given the wide array of interventions for the management of T2DM, decisions to fund and implement these should be informed by estimates of both efficacy and cost-effectiveness. This ensures that patients are provided with treatments that represent the optimal use of scarce resources.

There is growing interest in telephone coaching interventions for people with T2DM. As suggested by the Diabetes Telephone Coaching Study (DTCS) [2], these interventions may to improve the risk factor status and diabetes management practices of people with T2DM. The DTCS recruited 94 participants with poorly controlled T2DM (HbA1C greater than 7 %) from the Diabetes Clinic at St Vincent’s Hospital Melbourne, an Australian tertiary hospital. Participants were randomised to usual care plus telephone coaching, or usual care alone for 6 months. Follow up occurred at 6 months (the end of the intervention period) and at 12 months (6 months after withdrawal of the intervention).

Diabetes coaching in this study was defined as the regular provision of telephone advice and coaching that addressed lifestyle modification, adherence to treatment schedules, goal setting and barriers to change. Specifically, monthly coaching sessions were delivered by a dietitian. Participants were encouraged to make changes to their diet and exercise habits; to discuss specific medication changes with their general practitioner (GP), and to adhere to the recommended schedule for foot checks, eye checks and vaccinations. Relevant goals were agreed upon at each coaching session and progress towards goal attainment was reviewed at subsequent coaching sessions. If goals were not achieved, barriers to goal attainment were identified and a plan that addressed these barriers was agreed. New goals were set as required. This process was repeated throughout the intervention.

The primary outcome, HbA1C at 6 months, was significantly lower among the intervention group compared to the controls, −0.8 %, 95 % confidence interval (CI) (−1.2 to −0.3) [2]. Other parameters that improved at 6 months included fasting glucose, diastolic blood pressure, physical activity and adherence to diabetes management practices. However, improvements observed at 6 months were not sustained at 12 months.

Although the DTCS did not show sustained benefits upon withdrawal of the coaching, numerous trials have indicated that the provision of ongoing follow-up and support facilitates the longer-term maintenance of intervention gains [3–12]. These trials strongly support the notion that if the telephone coaching was delivered on an ongoing basis, improvements observed at 6 months in the DTCS are likely to be maintained.

Extrapolating from the results of the DTCS, the present analysis sought to assess the cost-effectiveness of telephone coaching for patients with T2DM. While many telephone coaching trials have speculated regarding the potential cost-effectiveness of these interventions, few have measured changes in resource use [26, 45, 46], and fewer still have assessed cost-effectiveness [47, 48]. This is the first Australian study to assess the cost-effectiveness of telephone coaching in a population exclusively with T2DM. Measuring costs in an Australian context is important due to international differences in healthcare costs [13, 14]. Importantly, estimates of cost-effectiveness are relevant to funding decisions that facilitate the translation of research evidence into clinical practice.

## Methods

A cost utility analysis was undertaken to compare telephone coaching with usual care. Six-month outcome data from the DTCS were applied to the UKPDS Outcomes Model in order to predict marginal changes in risks of clinical events (myocardial infarction [MI], coronary heart disease [CHD], stroke, congestive heart failure [CHF], amputation, renal failure and blindness), years lived, quality-adjusted life years (QALYs) lived and costs. The analysis took a health system perspective, considering direct healthcare costs met by the Victorian State and Commonwealth Governments.

It was assumed that intervention group participants received telephone coaching for 10 years, with intervention costs maintained during each year that participants were predicted to survive. Although other telephone coaching trials have observed improved glycaemic control with 12 months of intervention [9, 15–20], and the maintenance literature indicates that the provision of ongoing follow-up and support facilitates the longer-term maintenance of intervention gains [3–12], the true effect of continuously delivering this intervention remains uncertain. Consequently, conservative assumptions were made concerning the impact of the intervention on HbA1C. Rather than assuming that HbA1C values observed at 6 months in the DTCS were maintained throughout the modelled time horizon, HbA1C values in each simulation year were predicted by the UKPDS model. Sensitivity analyses were also conducted to account for this uncertainty.

In the base-case analysis, a 5 % discount rate was applied to all future costs and benefits. This rate was varied in the sensitivity analyses to reflect uncertainty. A 10 year time horizon was chosen for the base-case analysis. This was varied in the sensitivity analyses to two, five and 15 years. The primary outcome was an incremental cost-effectiveness ratio (ICER), expressed as a cost per QALY saved.

The UKPDS Outcomes Model is a probabilistic, discrete-time computer simulation model that uses algorithms based on UKPDS data to predict the development of seven diabetes-related complications (MI, CHD, stroke, CHF, amputation, renal failure and blindness) and death. The model enables economic evaluations of interventions that affect risk factors in people with T2DM [21]. In the present analysis, model subjects comprised participants of the DTCS, who entered the model with characteristics based on levels at the end of the six month intervention period. Missing data at 6 months were imputed using the last observation carried forward method, with values observed at baseline used to impute missing values at 6 months. The model also demands data concerning the risk factor status of participants at diagnosis of T2DM. This information was not available to investigators, therefore, it was assumed that these levels were the same as those recorded at the participant’s baseline assessment in the DTCS. The model ran in one year cycles, for which the risks of complications and death were predicted. Predictions were made based on each participant’s six month characteristics and risk factors that the model changed with time. The model accounted for event-related dependencies, whereby the presence of one complication (such as CHD) increased the likelihood of another (such as CHF) and furthermore increased the risk of death. Participants continued through the model for 10 cycles, or until death.

Key model inputs are summarised in the Additional file 1. Health utility values were updated following each model cycle and used to calculate QALYs at the end of the simulation period. Multiple complications were assumed to have an additive effect on quality of life. The health utility values assigned to participants were based on UKPDS data [22].

Costs were reported in 2012/13 Australian dollars. Costs were deflated to their net present value using the Health Price Index [23]. The model applied acute and ongoing costs to events predicted to develop in the simulation period. These costs were sourced from Australian data [24].

A cost was also applied to participants without diabetes-related complications. This cost reflected diabetes-related costs incurred by DTCS participants between baseline and 6 months of the study, and thus considered the cost of medications, general practitioner presentations, St Vincent’s Hospital outpatient appointments, St Vincent’s Hospital emergency department presentations and St Vincent’s Hospital inpatient admissions. This six monthly cost was multiplied by a factor of two to estimate annual costs.

To account for the cost of the telephone coaching intervention, an annual discounted cost was applied to intervention group participants ‘post-hoc’. This reflected staffing and telephone call costs and was added to the cost of intervention group participants during each simulation year they were predicted to survive. One-way sensitivity analyses were conducted to reflect uncertainty surrounding key input parameters (Table 1).

**Table 1:** Parameters varied in sensitivity analyses

Key input parameter	Sensitivity analyses
Time horizon	Five, 15, 20 years
Discount rate	3 %, 4 %, 6 % to future costs and effects
Health utilities	Varied according to the upper and lower limits of the 95 % CI surrounding mean values reported by Clarke and colleagues (Clarke et al., 2002)
Cost of complications	Varied according to the upper and lower limits of the 95 % CI surrounding mean values reported by Clarke and colleagues (Clarke et al., 2008)
Cost in the absence of complications	Varied according to the upper and lower limits of the 95 % CI surrounding mean values reported in DTCS.
HbA1C	Assumed that HbA1C at 6 months in the DTCS was maintained for one, two and five simulation years.
Stroke	Assumed that no participants had a past history of stroke.

All procedures followed in this study complied with requirements of the St Vincent’s Hospital Human Research Ethics Committee.

## Results

The groups were balanced at entry into the model with the exception of HbA1C levels, these being lower in the intervention group, 7.8 % versus 8.7 %, *p* = 0.003 (reflecting the efficacy of the intervention delivered in the DTCS). In addition, intervention group participants were less commonly Asian/Indian and more commonly Caucasian. The groups differed in the number of years since they had suffered a stroke (Table 2). This difference reflected a finding from the DTCS showing that fewer intervention group participants had previously suffered a stroke, nil versus 8 (17 %). Based on data collected between baseline and 6 months of the DTCS, annual costs were applied to participants in each group to reflect the annual cost of treating participants without diabetes-related complications. The mean (95 % CI) costs applied to intervention and control group participants were $6091 (2183–9998) and $3107 (2530–3683), respectively. To reflect the cost of delivering the telephone coaching intervention, a cost of $1286 was applied to intervention group participants during each simulation year they were predicted to survive.

**Table 2:** Characteristics of the simulated population

		Intervention group, *n* = 47	Control group, *n* = 47	Total, *n* = 94	*P*-value
Demographic characteristics					
Ethnicity, n (%)	Caucasian	46 (98)	37 (79)	83 (88)	**0.02**
	Afro-Caribbean	0 (2)	2 (4)	2 (2)	
	Asian/Indian	1 (2)	8 (17)	9 (10)	
Gender, n (%)	Male	34 (72 %)	30 (64 %)	64 (68)	0.51
	Female	13 (28 %)	17 (36 %)	30 (32)	
Age at diagnosis (years)		47 (44–50)	50 (47–53)	48 (46–51)	0.13
Diabetes duration (years)		13 (10–15)	13 (11–16)	13 (11–15)	0.75
Risk factor values at diagnosis of T2DM					
AF, n (%)		1 (2)	0 (0)	1 (2)	1.00
Peripheral vascular disease n (%)		0 (0)	2 (4)	2 (4)	0.5
Smoking n (%)	Current smoker	18 (38)	17 (36)	35 (37)	0.57
	Never smoker	21 (45)	25 (53)	46 (49)	
	Ex-smoker	8 (17)	5 (11)	13 (14)	
Cholesterol (mmol/l)		4.1 (3.9–4.4)	4.5 (4.1–4.9)	4.3 (4.0–4.5)	0.15
High density lipoprotein (mmol/l)		1.1 (1.0–1.2)	1.2 (1.1–1.2)	1.1 (1.1–1.2)	0.42
Systolic blood pressure (mmHg)		140 (134–145)	134 (128–140)	137 (133–141)	0.13
HbA1c (%)		8.2 (8.0–9.7)	8.5 (8.1–8.9)	8.3 (8.1–8.6)	0.18
Risk factor values at entry into the model (6 months in the DTCS)					
Smoking, n (%)	Current smoker	5 (11)	8 (17)	13 (14)	0.3
	Never smoker	21 (45)	25 (53)	46 (49)	
	Ex-smoker	21 (45)	14 (29)	35 (37)	
Cholesterol (mmol/l)		4.0 (3.8–4.3)	4.5 (4.0–4.9)	4.3 (4.0–4.5)	0.07
High density lipoprotein (mmol/l)		1.1 (1.0–1.1)	1.2 (1.1–1.2)	1.1 (1.1–1.2)	0.12
Systolic blood pressure (mmHg)		133 (128–138)	132 (127–138)	133 (129–136)	0.9
HbA1C (%)		7.8 (7.4–8.1)	8.7 (8.2–9.2)	8.2 (7.9–8.5)	**0.003**
Years since pre-existing event					
CHD (excluding MI)		0.8 (0.0–1.6)	1.3 (0.2–2.4)	1.1 (0.4–1.7)	0.45
CHF		0.1 (0.0–0.3)	0.3 (0.0–0.6)	0.2 (0.0–0.4)	0.32
Amputation		0.1 (0.0–0.2)	0.0 (0.0–0.1)	0.1 (0.0–0.1)	0.4
Blindness		0.5 (0.0–1.1)	0.1 (0.0–0.3)	0.3 (0.0–0.6)	0.15
Renal failure		0.2 (0.2–0.6)	0.3 (0.0–0.5)	0.3 (0.0–0.5)	0.71
Stroke		0.0 (0.0–0.1)	1.3 (0.2–2.4)	0.7 (0.1–1.2)	**0.03**
MI		1.2 (0.3–2.2)	2.7 (0.4–5.0)	1.9 (0.7–3.2)	0.25

All results presented as mean (95 % CI) unless otherwise specified. *P* values in bold < 0.05 and considered statistically significant

**Fig. 1:**
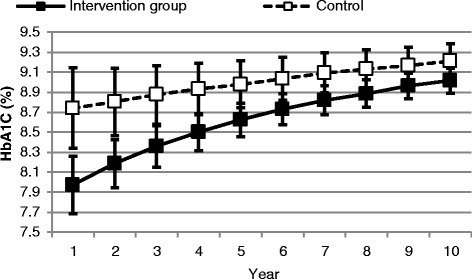
Change in mean (95 % CI) HbA1C over 10 years

**Fig. 2:**
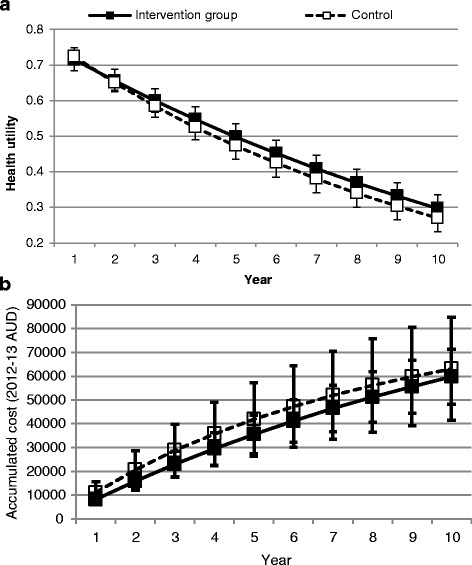
**a** Predicted change in mean (95 % CI) health utility over 10 years, and **b** Predicted change in mean (95% CI) cumulative costs over 10 years

Over 10 years, the model predicted that the intervention would dominate the comparator, contributing to net health benefits at a lower cost. The intervention contributed to savings of over $3300 per participant and an incremental gain of 0.2 QALYs. Ten year discounted costs were $59,790 and $63,117 among intervention versus control group participants, respectively, while 4.88 and 4.68 discounted QALYs were lived by the two groups. The intervention contributed to an incremental gain in life expectancy of 0.3 years (Table 3). The model predicted that the between-group difference in HbA1C at entry into the model reduced over time (Fig. 1). There was a trend toward higher health utility scores and lower annual treatment costs among intervention group participants (Fig. 2a and b).

**Table 3:** Findings from the base-case analysis

	Intervention group, *n* = 47	Control group, *n* = 47	Difference
Life expectancy (years)	8.1	7.7	0.3
Total QALE	4.9	4.7	0.2
Cost of the intervention ($)	8581	0	8581
Cost of complications ($)	51,210	63,117	−11,907
Total cost ($)	59,790	63,117	−3327

*ICER* intervention dominated the control condition

All results presented as a point estimate (mean)

Table 4 summarises the cumulative incidence of first events among participants in each group over 10 years. The 10 year risk of any complication was lower in the intervention group, 32 % versus 38 %. The risk of death was also lower among intervention group participants, 32 intervention group participants predicted to survive for 10 years compared to 30 controls.

**Table 4:** Mean (95 % CI) cumulative incidence of first events over 10 years

	Intervention group, *n* = 47		Control group, *n* = 47	
	Cumulative incidence	Number of cases	Cumulative incidence	Number of cases
CHD	0.05	2	0.05	2
MI	0.13	6	0.17	8
CHF	0.04	2	0.05	2
Stroke	0.05	2	0.05	2
Amputation	0.02	1	0.02	1
Blindness	0.02	1	0.03	1
Renal failure	0.02	1	0.01	0
Any complication	0.32	15	0.38	18
Diabetes-related death	0.12	6	0.17	7
All death	0.36	15	0.43	17

The intervention dominated the control condition under most conditions tested in sensitivity analyses. The largest savings were observed when the treatment costs of participants without complications were adjusted to reflect the lower limit of the 95 % CI surrounding this value (Table 5).

**Table 5:** Findings from the sensitivity analyses

		Quality adjusted life expectancy (QALYs)			Direct healthcare costs (2012/13 Australian dollars)			ICER (Cost per QALY)
		Intervention group (*n* = 47)	Control group (*n* = 47)	Difference	Intervention group (*n* = 47)	Control group (*n* = 47)	Difference	
Base-case		4.9 (4.5–5.2)	4.7 (4.4–5.0)	0.2 (0–0.3)	59,790 (48,182–71,399)	63,117 (41,490–84,745)	−3327 (−27,645–20,991)	Dominant
Time horizon	Two years	1.4 (1.3–1.4)	1.4 (1.3–1.4)	0.0 (−0.1–0.1)	16,581 (12,619–20,543)	20,440 (12,436–28,445)	−3859 (−12,715–4997)	$3859 saved Nil benefit
	Five years	3.1 (2.9–3.2)	3.0 (2.8–3.1)	0.1 (−0.2–0.3)	36,801 (28,599–45,003)	41,562 (26,330–56,795)	−4761 (−21,901–12,378)	Dominant
	15 years	6.9 (6.2–7.5)	6.5 (5.8–7.1)	0.4 (−0.5–1.3)	85,014 (70,189–99,838)	85,124 (57,566–112,681)	−110 (−31,111–30,890)	Dominant
Discount rate	0 %	6.2 (5.8–6.7)	5.9 (5.5–6.4)	0.3 (−0.3–0.9)	73,839 (59,390–88,288)	78,987 (52,235–105,739)	−5148 (−35,268–24,972)	Dominant
	3 %	5.4 (5.0–5.7)	5.2 (4.8–5.5)	0.2 (−0.3–0.8)	65,125 (52,499–77,750)	68,797 (45,334–92,261)	−3673 (−30,070–22,724)	Dominant
	6 %	4.7 (4.4–5.0)	4.5 (4.2–4.8)	0.2 (−0.3–0.6)	56,800 (45,654–67,946)	60,556 (39,758–81,354)	−3756 (−27,134–19,622)	Dominant
Utility scores	Lower limit of 95 % CI	4.8 (4.5–5.2)	4.6 (4.3–5.0)	0.2 (−0.3–0.7)	59,790 (48,182–71,399	63,117 (41,490–84,745)	−3327 (−27,645–20,991)	Dominant
	Upper limit of 95 % CI	4.9 (4.6–5.2)	4.7 (4.4–5.0)	0.2 (−0.3–0.7)	59,790 (48,182–71,399	63,117 (41,490–84,745)	−3327 (−27,645–20,991)	Dominant
Cost of complications	Lower limit of 95 % CI	4.9 (4.5–5.2)	4.7 (4.4–5.0)	0.2 (0–0.3)	53,979 (45,646–62,311)	52,493 (36,526–68,460)	1485 (−16,285–19,256)	$7425 per QALY
	Upper limit of 95 % CI	4.9 (4.5–5.2)	4.7 (4.4–5.0)	0.2 (0–0.3)	66,455 (51,397–81,513)	74,526 (47,419–101,633)	−8071 (−38,667–22,524)	Dominant
Cost – no complications	Lower limit of 95 % CI	4.9 (4.5–5.2)	4.7 (4.4–5.0)	0.2 (−0.3–0.7)	45,347 (32,674–58,020)	61,116 (39,199–83,034)	−15,769 (−40,833–9294)	Dominant
	Upper limit of 95 % CI	4.9 (4.7–5.1)	4.7 (4.4–4.9)	0.2 (−0.3–0.7)	74,261 (62,640–85,881)	65,139 (43,796–86,483)	9121 (−14,951–33,194)	$45,605 per QALY
HbA1C	HbA1C maintained for one simulation year	4.9 (4.7–5.1)	4.7 (4.4–4.9)	0.2 (−0.3–0.7)	59,931 (48,276–71,586)	63,214 (41,475–84,953)	−3283 (−27,721–21,155)	Dominant
	HbA1C maintained for two simulation years	4.9 (4.7–5.1)	4.7 (4.4–4.9)	0.2 (−0.3–0.7)	59,986 (48,307–71,665)	62,628 (41,116–84,140)	−2642 (−26,890–21,606)	Dominant
	HbA1C maintained for five simulation years	4.9 (4.7–5.1)	4.7 (4.4–4.9)	0.2 (−0.3–0.7)	60,014 (48,343–71,685)	62,974 (41,251–84,696)	−2960 (−27,390–21,470)	Dominant
Stroke	Nil past history of stroke in either group	4.9 (4.5–5.2)	4.7 (4.3–5.0)	0.2 (−0.3–0.7)	59,134 47,497–70,771	58,261 (38,557–77,966)	873 (−21,778–23,523)	$4365 per QALY

Mean (95 % CI) unless otherwise specified

## Discussion

The results of these analyses suggest that the cost of investing in telephone coaching would be fully recovered through cost savings over 10 years. Treatment costs were $3327 lower among intervention group participants. Savings were driven by lower costs associated with treating diabetes-related complications; the cost of treating these was almost $12,000 lower per intervention group participant. Intervention group participants also gained an additional 0.20 QALYs and 0.3 years of life over 10 years. Like cost savings, improvements in QALE and life expectancy were driven by reductions in the risk of complications. The 10 year risk of MI, CHF, any complication and death was lower among intervention group participants, with risk reductions of 24, 20, 13 and 16 % observed. Given that the DTCS was powered for the primary endpoint of change in HbA1C, it is likely that a much larger sample size would be required to demonstrate statistical significance for the economic analysis. Nevertheless, an intervention which would save over $3000 per patient over 10 years would result in substantial cost reductions across the health care system, even if the clinical endpoints were neutral.

Predicted cost savings and net health benefits were apparent despite conservative assumptions concerning the intervention’s cost and its impact on glycaemic control. For instance, rather than assuming that HbA1C levels at 6 months in the DTCS were sustained in subsequent simulation years, trends in HbA1C were predicted by the model. Consequently, glycaemic control was predicted to deteriorate in both groups over time. Costs were also applied conservatively, with higher annual treatment costs applied to the intervention group to account for the cost of the telephone coaching intervention and other treatment costs that were higher in this group during the trial. Having applied these conservative assumptions, confidence in the validity of this study’s findings is further enhanced.

Also enhancing confidence in the validity were results showing that predictions of cost savings were robust to most conditions tested in the sensitivity analyses. The greatest cost savings were observed when cost of treating participants without diabetes-related complications was adjusted to reflect the lower limit of the 95 % CI surrounding this value. However, cost savings disappeared when past history of stroke was controlled for, suggesting that this chance imbalance between the groups may have biased findings in favour of the intervention group. However, at a cost of $4365 per QALY, the intervention was considered highly cost-effective under this condition and therefore, should still prove attractive to decision makers considering whether this intervention should be implemented into routine clinical practice.

Only one Australian study was identified as having assessed the cost-effectiveness of a telephone delivered, behaviour change counselling intervention in people with T2DM. The analysis drew upon data from a randomised controlled trial (RCT) which found that a 12 month telephone coaching intervention contributed to significant improvements in diet but not physical activity in participants with T2DM or hypertension [25]. Modelled over 10 years and compared with usual care, the intervention was not cost-effective, however, compared with existing practice, the intervention was considered cost-effective at a cost of $29,375 per QALY gained [26]. Investigators in this study differentiated existing practice from usual care, noting that participants receiving usual care received more intervention (telephone calls for data collection, verbal feedback on dietary and exercise behaviour and written education material) than was typical under existing practice conditions.

The present economic analysis might be differentiated from that of Graves and colleagues in a number of respects. For instance, the RCT on which Graves and colleagues’ economic analysis was based, recruited participants with either T2DM or hypertension [25]. Therefore, projections of costs and effects do not relate specifically to people with T2DM. Furthermore, the study extrapolated outcomes observed at 12 months in the RCT (namely the intervention’s impact on physical activity) to predict cost-effectiveness over 10 years [25]. However, physical activity is less reliable as a marker of long-term outcomes in T2DM than HbA1C. Whereas prospective RCTs demonstrate a cause and effect relationship between HbA1C, morbidity and mortality (key drivers of costs and effects in people with T2DM), evidence concerning the impact of physical activity on such endpoints comes from epidemiological and cohort studies [27–30]. Therefore, the present economic analysis may provide a more reliable estimate concerning the cost-effectiveness of telephone coaching in people with T2DM.

No other telephone coaching trials were identified as having contributed to both cost savings and net health benefits in people with T2DM. However, comparison of findings from the present economic analysis with other telephone coaching studies is difficult, firstly, because most were conducted in other countries and secondly, because of methodological issues that limit the validity and generalisability of their findings. For instance, one study expressed the ICER as a cost per unit change in a surrogate endpoint [31], another measured only costs [32] and several conducted only within-trial economic analyses, failing to project outcomes over a sufficient time horizon to facilitate valid comparison with findings from this economic analysis [31–35]. Comparison with results from other countries is invalid owing to international differences in health systems and healthcare costs [13, 14]. Therefore, this economic analysis makes a valuable contribution to knowledge concerning the cost-effectiveness of telephone coaching in Australians with T2DM.

As with all modelling analyses, a degree of uncertainty surrounds predictions obtained through the extrapolation of data from a short-term clinical trial that never empirically assessed the intervention’s impact on survival, event rates, costs or QALE. For instance, confounding may have been present due to the age difference between the intervention and comparator groups, but having randomised the groups in the original study, this is unlikely to have changed the conclusion that the DTCS would likely be highly cost-effective. Longer, prospective RCTs are required to validate predictions obtained in this study. Simulation models provide a parsimonious solution to the absence of such long-term prospective data and despite their limitations, are widely used to extrapolate outcomes beyond the conclusion of clinical trials. Having applied conservative assumptions, the best available simulation model and extensive sensitivity analyses, the validity of findings from this study might be enhanced. As our evidence is indirect, caution should be taken in interpreting the results.

Limitations also relate to the UKPDS Outcomes Model. Previous studies have indicated that this model over-estimates event rates and mortality risk in populations dissimilar to the one on which it was developed [36, 37]. The model has not been validated for use in an Australian population which is multicultural. Furthermore, the model predicts only a limited range of complications and predicts only first, not subsequent events [21]. However, given that no other simulation models have been validated for Australian populations, this model was considered the best available for the purpose of this economic analysis [38].

Other limitations relate to the measurement of costs and effects. Consistent with a health system perspective, only direct diabetes-related costs were considered. Therefore, societal costs (to individuals and carers through lost time, income and productivity) were not captured. In terms of effects, utility weights applied in this study were not determined empirically, but were instead sourced from the literature. It is likely that these values would differ from those that would be obtained had DTCS participants been surveyed directly.

Findings from this analysis should be considered transferable to Australians with long-standing, T2DM that is sub-optimally controlled. This population is substantial; self-reported data from 2007 to 08 have indicated that 3.8 % of Australians (787,500 people) are affected by T2DM [39] and observational data have indicated that poor glycaemic control is common among Australians with T2DM [40–43].

## Conclusions

Interest in diabetes coaching interventions is growing, with numerous such studies listed on the Australian New Zealand Clinical Trials Registry, many of which are collecting real-time, cost-effectiveness data. The RCT on which the present economic analysis was based found that adding a six month telephone coaching intervention to the usual care regimen of participants with poorly controlled T2DM led to improvements in glycaemic control and a range of other parameters. This economic analysis has shown that under conditions of the base-case analysis and most sensitivity analyses, the intervention would contribute to net health benefits and cost savings. In assessing cost-effectiveness, this study has extended findings from the existing telephone coaching literature. Two sensitivity analyses did not predict cost savings, instead predicting that the intervention would be highly cost-effective at a cost of less than $10,000 per QALY. It has previously been stated that dominant interventions and interventions that cost less than $10,000 per QALY should only be ignored if ‘decision-makers have very serious reservations about the evidence base or are facing insurmountable problems in relation to stakeholder acceptability or feasibility of implementation’ [44]. Findings from this study support the need for a longer, prospective multi-centre trial of telephone coaching to confirm both the clinical and economic benefits prior to implementation into routine clinical practice. Future research should also consider alternative coaching delivery methods, using online and mobile interactive tools.

## Additional file

Additional file 1: Key input data for the UKPDS Outcome Model. (DOCX 21 kb)

## Abbreviations

CHD: Coronary Heart Disease
CHF: Congestive Heart Failure
CI: Confidence Interval
DTCS: Diabetes Telephone Coaching Study
ICER: Incremental Cost-Effectiveness Ratio
MI: Myocardial Infarction
QALY: Quality-Adjusted Life Years
RCT: Randomised Controlled Trial
T2DM: Type 2 Diabetes
UKPDS: United Kingdom Prospective Diabetes Study
