# Supplementary material for: The cost-effectiveness of hospital-based telephone coaching for people with type 2 diabetes: a 10 year modelling analysis
Source: BMC Health Serv Res. 2016 Sep 27;16:521. doi: 10.1186/s12913-016-1645-6 (PMC5039787; doi:10.1186/s12913-016-1645-6)
Supplement: Additional file 1: — Key input data for the UKPDS Outcome Model. (DOCX 21 kb) [file 12913_2016_1645_MOESM1_ESM.docx]

Key model inputs are summarised in Table 1. Health utility values used by the model were based on published data[[22](#_ENREF_22)]. Therefore, a health utility score of 0.78 was applied to participants without diabetes complications. Utility decrements applied to participants with a history of complications are summarised in Table 2. Cost of treating participants with and without diabetes related complications and of delivering the intervention in each simulation year are summarised in Tables 3-5, respectively.

Table 1: Key input data required by the UKPDS Outcomes Model

| **Demographic characteristics:** | Ethnicity (Afro-Caribbean, Caucasian, Asian/Indian)  Gender (male/female)  Age at diagnosis of T2DM (years)  Diabetes duration (years)  Weight (kg)  Height (cm) |
| --- | --- |
| **Risk factor values at diagnosis of T2DM** | AF (yes/no)  PVD (yes/no)  Smoking (current/never/ex-smoker)  Cholesterol (mmol/l)  HDL (mmol/l)  Systolic BP (mmHg)  HbA1C (%) |
| **Current risk factor values** | Smoking (current/never/ex-smoker)  Cholesterol (mmol/l)  HDL (mmol/l)  Systolic BP (mmHg)  HbA1C (%) |
| **Years since pre-existing event** | CHD - excluding MI (years)  CHF (years)  Amputation (years)  Blindness in one or both eyes (years)  Renal failure (years)  Stroke (years)  MI (years) |

Table 2: Utility decrements applied to participants during the simulation period

| **Complication** | **Utility decrement** |
| --- | --- |
| **CHD** | -0.09 (-0.126- -0.054) |
| **MI** | -0.055 (-0.067- -0.042) |
| **CHF** | -0.108 (-0.169- -0.048) |
| **Stroke** | -0.164 (-0.222- -0.105) |
| **Amputation** | -0.28 (-0.389- -0.170) |
| **Blindness** | -0.074 (-0.252- -0.124) |
| **Renal failure** | -0.263 (-0.263- -0.263) |

**Table 3: Annual cost of diabetes-related complications during the year the complication developed and in subsequent years**

|  | **In year complication develops** | | **In subsequent years** |
| --- | --- | --- | --- |
|  | **Fatal** | **Non-fatal** | **Cost** |
| **CHD** | - | 20249  (19362-21128) | 3429  (3007-3862) |
| **MI** | 15443  (12065-18972 ) | 18773  (17599-20045) | 2706  (2454-2979) |
| **CHF** | 25003  (22484-27384) | 25003  (22484-27384) | 10462  (8298-13302) |
| **Stroke** | 22807  (14633-31583) | 22559  (20759-24445) | 5943  (5194-6810) |
| **Amputation** | 32870  (30059-36082) | 32870  (30059-36082) | 6490  (5092-8087) |
| **Blindness** | - | 14297  (10262-20360) | 4893  (2557-7506) |
| **Renal failure** | 46144  (37012-55065) | 46144  (37012-55065) | 49044  (36125-60961) |

***All costs reported in $AU 2012/13.**

Table 4: Annual diabetes-related costs of participants without complications in the intervention versus control groups

|  | **Intervention**  **(n=25)** | **Control**  **(n=22)** |
| --- | --- | --- |
| **Diabetes-related costs** | 6091  (2183-9998) | 3107  (2530 – 3683) |

***All costs reported in $AU 2012/13.**

Table 5: Annual discounted cost of the telephone coaching intervention in the first through to the 10^th^ simulation year

| **Year** | **Discounted cost** |
| --- | --- |
| Year one | 1286 |
| Year two | 1225 |
| Year three | 1166 |
| Year four | 1111 |
| Year five | 1058 |
| Year six | 1008 |
| Year seven | 960 |
| Year eight | 914 |
| Year nine | 870 |
| Year 10 | 829 |

***All costs reported in $AU 2012/13.**
